# Tying a knot between crown ethers and porphyrins

**DOI:** 10.3762/bjoc.19.120

**Published:** 2023-10-27

**Authors:** Maksym Matviyishyn, Bartosz Szyszko

**Affiliations:** 1 Faculty of Chemistry, University of Wrocław, 14 F. Joliot-Curie St., 50-383 Wrocław, Poland

**Keywords:** crown ethers, porphyrinoids, self-assembly, sensors, supramolecular chemistry

## Abstract

Porphyrins and crown ether hybrids have emerged as a promising class of molecules composed of elements of a tetrapyrrole macrocycle and an oligo(ethylene glycol) segment. These hybrid systems constitute a broad group of compounds, including crowned porphyrins, crownphyrins, and calixpyrrole-crown ether systems forming Pacman complexes with transition metals. Their unique nature accustoms them as excellent ligands and hosts capable of binding guest molecules/ions, but also to undergo unusual transformations, such as metal-induced expansion/contraction. Depending on the design of the particular hybrid, they present unique features involving intriguing redox chemistry, interesting optical properties, and reactivity towards transition metals. In this perspective article, the overview of both the early designs of porphyrin-crown ether hybrids, as well as the most recent advances in the synthesis and characterisation of this remarkable group of macrocyclic systems, are addressed. The discussion covers the strategies employed in synthesising these systems, including cyclisation reactions, self-assembly, and their remarkable reactivity. The potential applications of porphyrin-crown ether hybrids are also highlighted. Moreover, the discussion identifies the challenges associated with synthesising and characterising hybrids, outlining the possible future directions.

## Introduction

Many areas of modern supramolecular chemistry, organic, inorganic, materials and coordination chemistry, are based upon macrocyclic compounds of specifically-designed structures and tailored functions [1–3]. The design of novel macrocyclic compounds has laid the ground for phenomenal developments constituting supramolecular chemistry as a branch of chemical sciences. These fundamental developments included the discovery of crown ethers by Pedersen [4], followed by the synthesis of three-dimensional cryptands by Lehn [5], and spherands by Cram [6]. Later on, various classes of macrocyclic compounds were designed, demonstrating remarkable features in areas spreading from simple coordination chemistry [7], through host–guest chemistry, sensing [8], biomedicine [9], and materials science [10].

These two classes of molecules seem particularly remarkable among the endless family of macrocyclic compounds due to their unique features and easy accessibility. The popularity of porphyrins and crown ethers has been so extensive that the whole range of these macrocycles is even commercially available.

**Figure 1:**
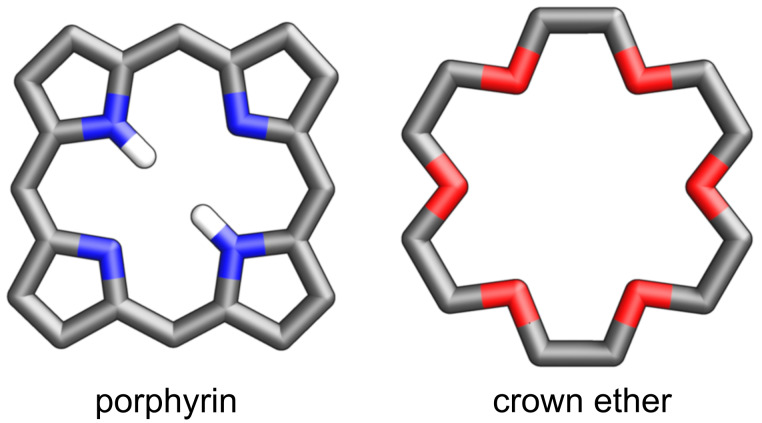
Porphyrin and crown ether.

Regarding the molecular design and their properties, porphyrins and crown ethers are like water and fire – they constitute the opposite elements (Figure 1). Porphyrins are built of four pyrrole rings, two of which are considered amine-like due to the presence of NH groups, whereas the other two have imine character [11–12]. They are rigid, planar molecules which feature macrocyclic aromaticity due to the cyclic delocalisation of π electrons [13]. The well-defined macrocyclic cavity termed the coordination core, can encompass one, two, or more central ions (typically metal/metalloid cations), forming coordination compounds wherein the central ion acquires a square planar [14], square pyramidal [15], or an octahedral coordination environment [16].

On the opposite side, crown ethers are cyclic molecules composed of carbon and oxygen atoms forming the macrocycle. Depending on the size of the ring and incorporated entities, they can present relative rigidity or flexibility [17]. The adaptable molecules of crown ethers render them excellent hosts for a wide range of alkali- or alkaline earth metals and organic guests with which they typically interact through hydrogen bonding/electrostatic interactions [18–20]. Replacing oxygen atoms with other elements, such as nitrogen, sulfur, etc., alters the crown ethers' affinity toward cations, extending their role as macrocyclic ligands to transition metals [21]. One can say that in many aspects, porphyrins and crown ethers are opposites, and as opposites attract, several studies were devoted to investigating the systems combining such contradictory structural elements [22–25].

In this perspective article, the subjective selection of literature demonstrating developments in the area of hybrid porphyrin-crown ether macrocycles is shown. As several topics were reviewed earlier, we did not intend to present an exhaustive overview but instead focused on presenting selected examples of molecules of fundamental importance in this research area. The more interested readers are referred to excellent review articles focused on specific classes of macrocycles, such as crowned porphyrins [22], calixpyrroles [26–29], Schiff porphyrinoids [30], expanded porphyrinoids [31–32], or carbaporphyrinoids [33–34], and a selection of articles dedicated to crown ether chemistry [17,20,35–37].

## Historical Perspective

**Figure 2:**
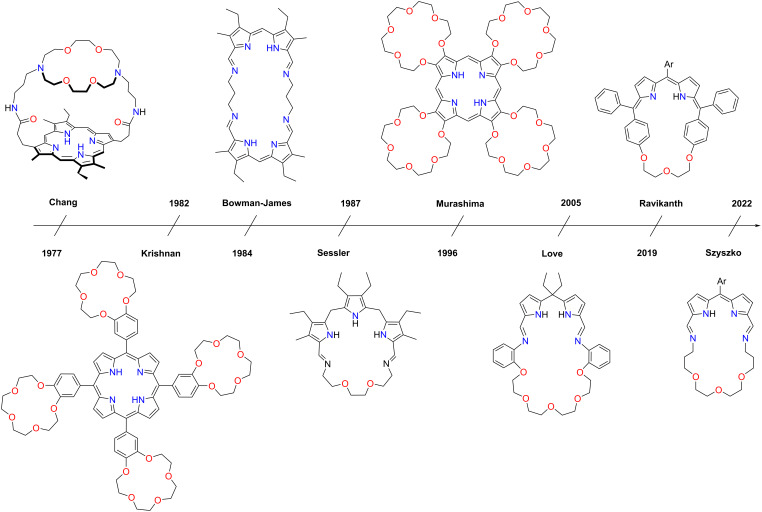
Timeline demonstrating the contributions into the crown ether–porphyrin chemistry.

Various macrocyclic compounds have been developed over the years where the segments of porphyrin and a crown ether were merged, forming a single chemical entity (Figure 2). In principle, they can be divided into two separate groups. The first one includes porphyrins substituted with crown ether at the peripheries of the molecule, i.e., at the β- or *meso*-positions. The other is constituted by molecules wherein the principal structural element of the porphyrin framework is connected with the oligo(ethylene glycol) chain forming the macrocycle. The examples briefly referred to in this chapter will be described in greater detail in the next sections.

In 1977 Chang provided the first example of a capped (strapped) β-pyrrole appended crown ether porphyrin. This result laid the foundation for future work on capped porphyrins [38]. The later contributions to the porphyrin macrocycle incorporating the crown ether motif date back to 1982 when Krishnan and Thanabal reported synthesising a new host molecule with multiple cavities capable of encompassing several guest molecules/ions [39]. The molecule demonstrated an exciting feature of binding Na^+^, Mg^2+^, Ca^2+^, K^+^, NH_4_^+^, and Ba^2+^ cations. The incorporation of cations such as K^+^, NH_4_^+^, and Ba^2+^ required two crown ether cavities attached to the porphyrin to form the coordination complexes through the dimerisation of the macrocycle. The dimensions of the crown ether pocket determined the complex formation; for example, if there was a mismatch in the sizes of the cation and crown ether pocket, dimerisation of the crown porphyrin molecule would occur. The dimerisation led to interesting changes in the visible, NMR, ESR, and emission spectral features. Further developments by Camilleri, Gunter, Boitrel, and Osuka focused on the exploitation of *meso*-crowned porphyrins as multitopic receptors, sensors, and supramolecular hosts, with applications in ion transport, catalysis, and polymeric materials [22,40–45].

In 1984 Lehn, Sessler and co-workers developed double-side-strapped crowned porphyrins, which served as tritopic and tetratopic receptors [46]. The macrotetracycles and macropentacycles, apart from the apparent metal complexation within the porphyrin core, showed cationic guest binding upon adding different ammonium salts, forming 1:1 complexes. Later, in 1985, Camilleri and co-workers reported the synthesis of a capped porphyrin macrocycle with a crown ether segment attached through the β-positions of the two pyrrole rings located oppositely to each other, hovering above the porphyrin plane [40]. The initiation of these research topics was crucial for the search of simple chemical models for haemoglobins and cytochromes, as well as multitopic receptors capable of binding ions and ion-pairs, which have been used in ion binding and catalysis, to name a few applications [47–49].

A primary example of a crown ether-annulated porphyrin, i.e., β-crowned porphyrin, was established in 1996 by Murashima and co-workers [50]. The macrocycle incorporated four pyrrole rings functionalised at their β positions with 18-crown-6 pockets. The research sparked interest in crown ether-annulated porphyrins to be used as potential multitopic chromophores, with the conjoined porphyrin and crown ether frameworks for electron transfer systems [51].

Regardless of the research on crowned porphyrins, the Bowman-James group worked on developing a new type of porphyrinoids called accordion porphyrins. Their seminal contribution, published in 1984, demonstrated a facile synthesis of an imine-linked tetrapyrrole macrocycle in the presence of a metal template and laid the ground for a whole new research area of hybrid imine-porphyrinoids [52–54]. Bowman-James' work provided an exciting insight into accordion porphyrins' coordination chemistry, demonstrating the tremendous potential of iminoporphyrinoids as macrocyclic ligands.

Independently the Sessler group worked on the alternative approach toward flexible iminoporphyrinoids built from tripyrrane [55–56]. They designed and synthesised the first porphyrinoid connecting the porphyrin and crown ether motifs within the macrocyclic framework [56]. This work laid a foundation for constructing crown ethers and tetrapyrrole hybrids, even though the macrocycle corresponded to porphyrinogen rather than the porphyrin framework. Sessler and co-workers dwelled on this discovery and introduced numerous examples of imine-based porphyrinoids, including texaphyrins [56–65].

The field remained practically unexplored until 2005 when the Love group reported Schiff-base calixpyrrole macrocycles introducing crown ether segments [66–67]. The hybrid systems were demonstrated to form a plethora of coordination compounds with transition metals. In 2019 Ravikanth developed another group of hybrid macrocycles in which the dipyrrin is connected to oligo(ethylene glycol) through carbon–carbon bonds [68]. In 2022, our group demonstrated the synthesis and reactivity of crownphyrins – hybrid macrocycles wherein the dipyrrin segment links with the crown ether part through the imine bonds [69].

## From Crowned Porphyrins to Crownphyrins

Several types of compounds merging the architecture of porphyrin and crown ether macrocycles were developed. These include *meso*- and β-crowned porphyrins and tetrapyrroles wherein the crown ether forms a strap on a single or both sides of the macrocycle. Furthermore, the formal replacement of a part of the porphyrin macrocycle with oligoethylene glycol opens further routes for constructing hybrid systems. This section will discuss syntheses, characterisation, and applications of various classes of crown ether–porphyrin architectures.

### Crowned porphyrins

Crowned porphyrins (or crown porphyrins) constitute a group of porphyrin macrocycles incorporating crown ether moieties introduced as a substituent at the *meso*- or β-position of the pyrrole ring(s) [22,70–71]. As this class of molecules was comprehensively reviewed earlier by Boitrel [22], we will only refer to selected examples demonstrating versatile applications of this class of macrocyclic receptors.

The interest in crowned porphyrins stems from their ability to selectively bind cations [72], acting as polytopic receptors and remarkable ligands. They were exploited in constructing supramolecular architectures [45], e.g., catenanes [73], rotaxanes [74], and catalytic systems [75]. Pelegrino and co-workers reported on crowned porphyrinoids demonstrating interesting photophysical properties [71]. The crown ether part was also demonstrated to play a role of a linker between two porphyrin macrocycles in systems designed for reaction-centre-like processes and their usage as potential energy transduction devices [51,76–77].

#### *meso*-Crowned porphyrins

**Figure 3:**
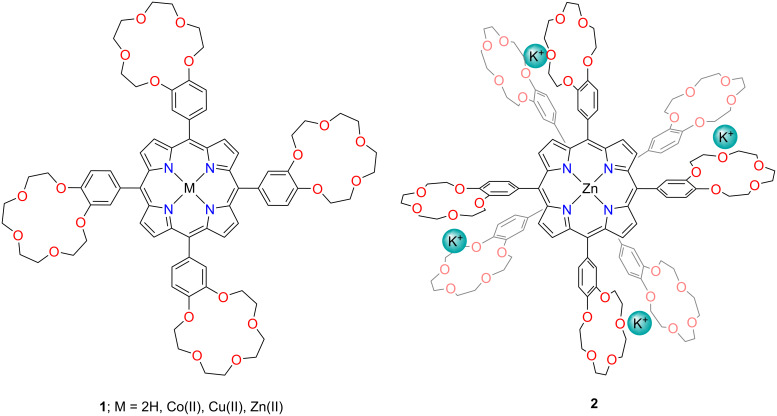
Tetra-crowned porphyrin **1** and dimer **2** formed upon K^+^ binding.

One of the conceptually most straightforward methods to introduce a crown ether unit into the porphyrin is functionalising the *meso*-substituents (Figure 3). *meso*-Crowned porphyrins contain a *meso*-bridged linkage between the crown ether moiety and the porphyrin macrocycle. Krishnan and Thanabal synthesised the first compounds of this type in 1982 [39]. The authors demonstrated a series of porphyrins incorporating a single (**MCP**, mono-crowned porphyrin), two (**DCP**), three (**TriCP**), and four benzo[15]crown[5] units (**TCP**, **1**).

The macrocycles belong to the interesting group of receptors showing binding to various guest cations, as the porphyrin cavity is typically best-fit for *d*-series cations, whereas the crown ether pocket is ideally suited for alkali metals. Due to the presence of crown ether moieties, the receptors exhibited selective binding of K^+^, NH_4_^+^, and Ba^2+^. The pronounced changes in the absorption spectra of free-base ligands **1** and **TriCP** with adding K^+^, NH_4_^+^, and Ba^2+^ cations were observed, whereas Na^+^, Mg^2+^, and Ca^2+^ did not produce appreciable effects. Furthermore, zinc(II) porphyrins **1-Zn** and **TriCP-Zn** bound potassium cations forming dimeric assemblies **2**, as demonstrated by the absorption and ^1^H NMR spectra. Although **DCP-Zn** and **MCP-Zn** were also capable of forming dimers, the highest stability and the largest level of attraction between the two monomers were expected for tetra-crowned **1-Zn**, where four potassium cations cooperatively clasp each monomer together. In such a case, K^+^ cations would ideally hover between the macrocyclic pockets of the crown ether moieties of each **1-Zn** monomer. Incorporation of other metals, e.g., Cu(II) and Co(II), into the porphyrin core introduced a probe, which enabled ESR spectroscopy methods as a means of the analysis of the assembled complexes with cationic guests bound within the crown ether pockets.

Over the years, several groups have shown interesting chemistry of *meso*-crowned porphyrins. Osuka and co-workers provided novel insight into the *meso*-appended crown ether porphyrins, namely, chromophore-incorporated and polymer-based systems capable of acting as multitopic receptors for transition, alkali, and alkali-earth metal cations, which were proved as optical sensing agents among other applications [43–44,78]. Fullerene-based crown ether-appended porphyrins developed by Diederich and co-workers were used as polymeric material films [79]. Intriguing supramolecular systems capable of electron transfer were developed by D'Souza, Ito and co-workers, showing selective multitopic receptors binding different alkali and transition metal cations with intramolecular photoinduced electron transfer [48,80]. Further applications of crown ether-appended porphyrins acting as multitopic receptors, catalytically active species, and ligands were also investigated [81–99].

**Figure 4:**
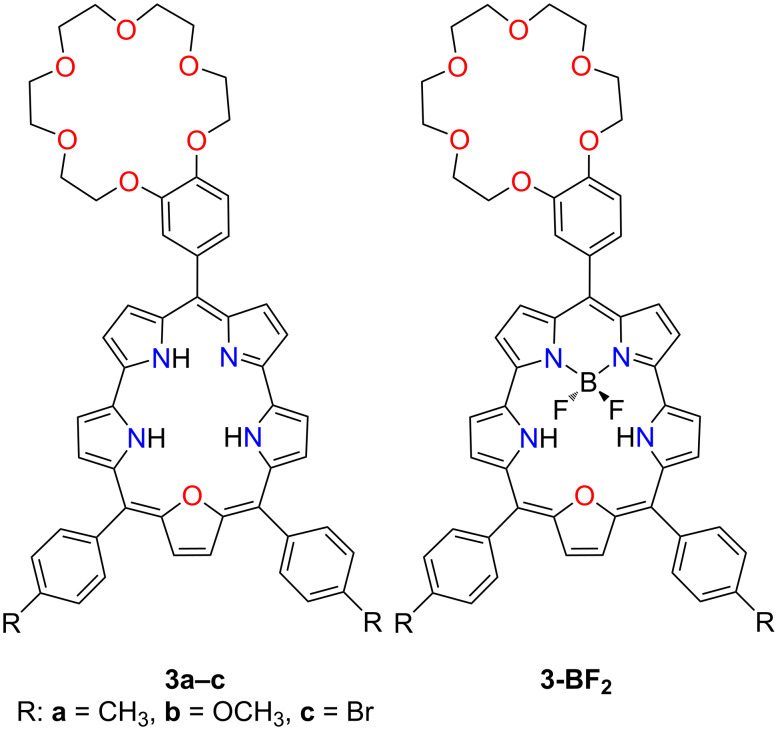
*meso*-Crowned 25-oxasmaragdyrins **3a–c** and their boron(III) complexes **(3a–c)-BF_2_**.

The Ravikanth group has developed a series of crown ether-appended expanded porphyrinoids, including 25-oxasmaragdyrins **3a–c** [100]. The macrocycles were demonstrated to form coordination compounds with boron(III) **3-BF_2_**, encompassing BF_2_ units coordinated to the dipyrrin part of the macrocycle (Figure 4). The introduction of the BF_2_ moiety into the free-base macrocycles consequently altered the fluorescent properties of the macrocyclic systems. The appended benzo-18-crown-6 in compounds **3a–c** was selective for K^+^, and the macrocycle fluorescence was slightly enhanced upon complexation.

#### Crowned calixpyrroles

**Scheme 1:**
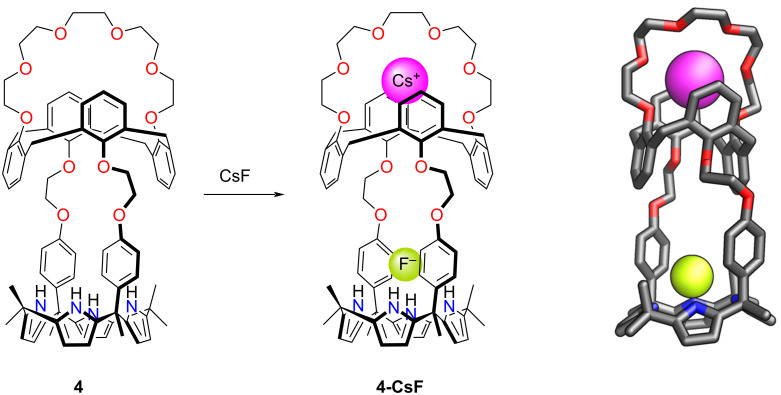
CsF ion-pair binding of **4**. The molecular structure of **4-CsF** is shown on the right [101].

In 2008, Sessler and co-workers synthesised an ion-pair receptor incorporating a calix[4]pyrrole framework functionalised with the crown ether units attached through *meso*-aryl groups (Scheme 1) [101]. The system comprised a crown-6-calix[4]arene-capped calix[4]pyrrole cavitand **4**. The heteroditopic receptor had multiple binding sites, proving efficient in encapsulating a CsF ion pair. The calix[4]arene-crown-6-capped pocket was exploited as an excellent binding site for the Cs^+^ cation, whereas the calix[4]pyrrole was aligned to trap the fluoride anion. The formed receptor: ion-pair 1:1 complex **4-CsF** was stable in solution, as evidenced by ^1^H NMR spectroscopy. The binding constant *K*_a_ = 3.8·10^5^ M^−1^ in CHCl_3_/MeOH 9:1 was reported. The XRD analysis in the solid state provided further proof of the binding mode, demonstrating the significant separation between the cation and anion.

**Figure 5:**
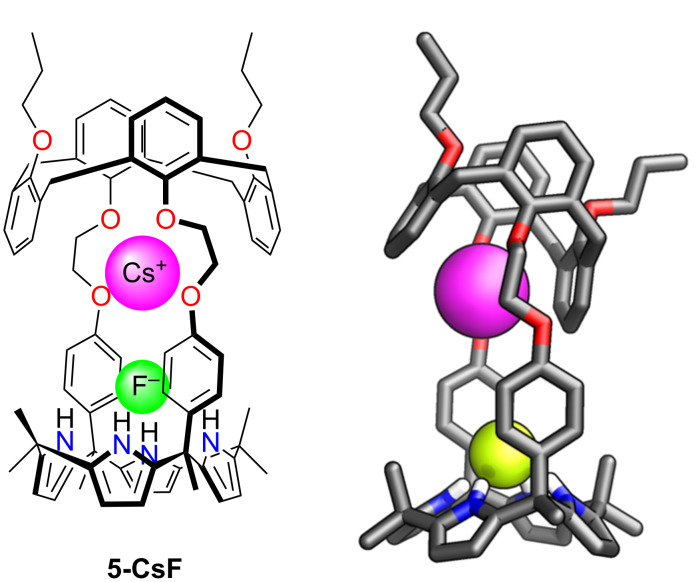
CsF ion pair binding by **5**. The molecular structure of **5-CsF** is shown on the right [102].

Sessler and co-workers introduced a similar ion-pair receptor, in which the calix[4]arene-strapped calix[4]pyrrole **5** demonstrated an additional binding mode of CsF (Figure 5). The binding constant *K*_a_ = 1.3·10^4^ M^−1^ in CHCl_3_/MeOH 9:1 was reported [101–102]. This included the binding of caesium cation in the oxygen-rich crown ether segment, with the fluoride interacting with NH of calix[4]pyrrole.

Additionally, receptor **5** formed an unprecedented 2:2 complex with CsCl, which included two different ion-pair binding sites, whereas with the addition of CsNO_3,_ a 1:1 complex was created, where both ions were held in close proximity. It was further reported that **5** could adapt its binding behaviour depending on the counteranion in the caesium salt.

**Scheme 2:**
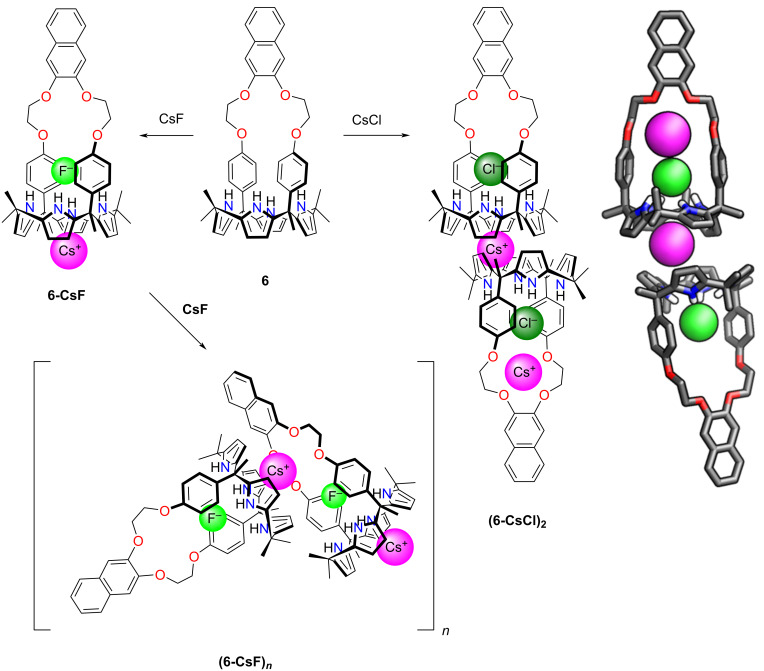
Ion-pair binding by **6**. The molecular structure of **(6-CsCl)_2_** is shown on the right [103].

Later Sessler and co-workers used naphthocrown-strapped calix[4]pyrrole **6** as a host to entrap CsF or CsCl ion pairs [103]. The CsF binding led to a supramolecular self-assembly process, inducing a sandwich host–guest complex formation in the solid state (Scheme 2). It was established that fluoride is preferred over any other halide anions. The binding of the ion pairs was observed in highly polar solvent media, but in the case of 10% methanol/chloroform, excess addition of CsF caused self-assembly into the sandwich host–guest. An analogous product **(6-CsCl)_2_** was demonstrated to form upon CsCl binding.

**Scheme 3:**
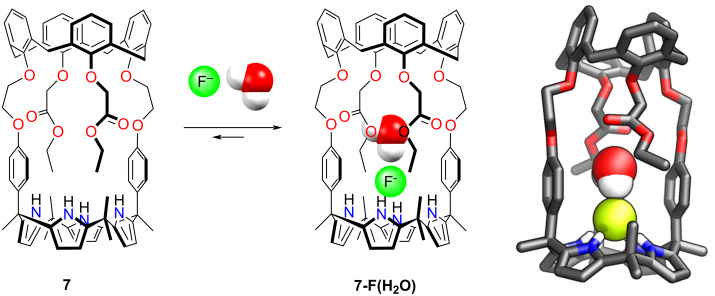
Hydrated fluoride binding by **7** [104].

An interesting behaviour was demonstrated for the calix[4]pyrrole-calix[4]arene receptor **7**, in which two macrocycles are linked through alkyl ether linkers, creating a crown ether-like core around the periphery of the calix[4]arene macrocycle (Scheme 3) [104]. The conformationally cone-locked receptor **7** showed the binding of monohydrated fluoride within the core. The F^−^ anion was encapsulated within the central cavity, interacting with the calixpyrrole macrocycle through hydrogen bonds. The water molecule was bound near the fluoride and was further stabilised through hydrogen bonding to the oxygen atoms in the central part of the receptor. This selective fluoride binding was evidenced with the help of ^1^H NMR spectroscopy. The addition of CsF showed an ion-pair host–guest complex formation.

#### β-Crowned porphyrins

**Figure 6:**
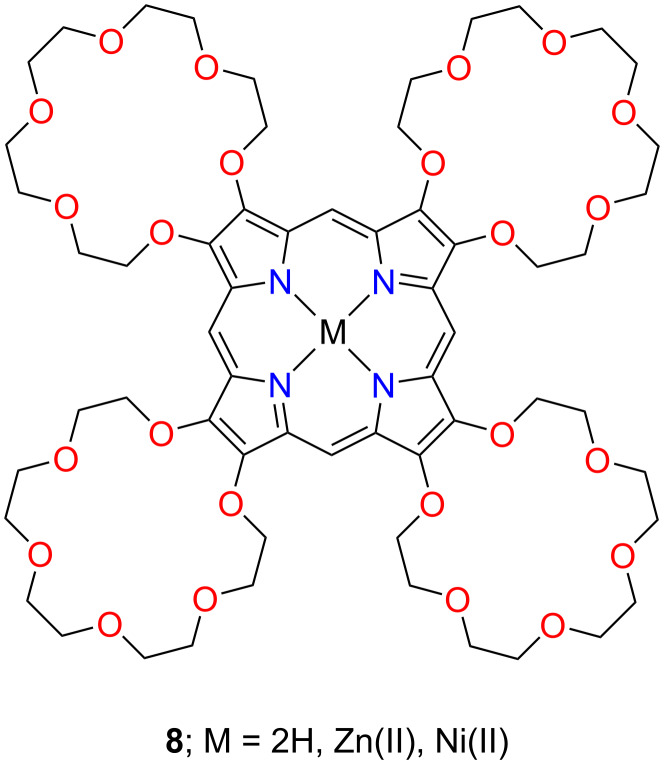
β-Crowned porphyrin **8**.

The β-crowned porphyrins can be considered a particular case of annulated tetrapyrroles [105]. This class of macrocycles incorporating β-substituted pyrrole rings can also be considered porphyrinocrown ethers, in analogy to benzocrown ethers. Murashima and co-workers synthesised a crown ether-annulated porphyrin **8** in 1996 [50]. The macrocycle contained a porphyrin core, with the eight β*-*positions substituted with four macrocyclic crown ether units (Figure 6). The tetraannulated compound **8**, having 18-crown-6 macrocyclic entities attached to pyrrolic subunits, acted as a receptor for alkali and alkali earth metal cations. Incorporating zinc(II) and nickel(II) into the porphyrin cavity yielded **8-Zn** and **8-Ni**.

Langford and co-workers developed an efficient method for synthesizing a series of porphyrin-appended crown ether systems, where the catechol unit of the crown ether was fused to two β-pyrrolic positions of the porphyrin periphery. The systems presented intriguing intramolecular electron-transfer properties. Additionally, they were investigated as fluorescent sensors for various organic and metal cations [51].

#### Crown ether-capped porphyrins

**Figure 7:**
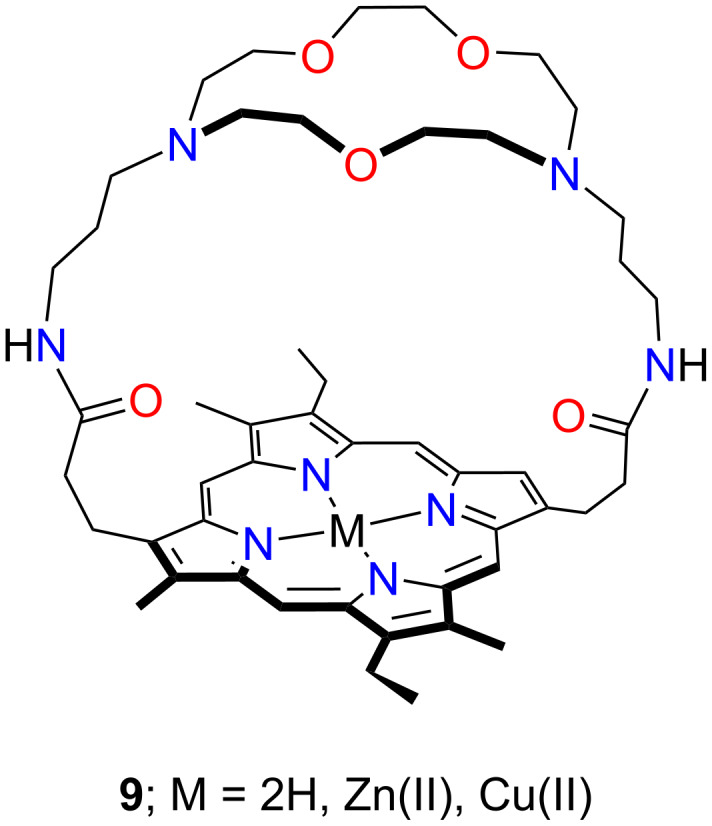
Crown ether-capped porphyrins **9**.

Crown ether capped β*-* and *meso*-porphyrins constitute hybrids comprising an ether ring attached to the tetrapyrrole, floating above and/or below the macrocyclic plane. In 1977 Chang developed the first example of a β-crown ether-capped porphyrin [38], providing a foundation for capped crown ether porphyrinoids. In 1985, Camilleri and co-workers described a macrocycle wherein the crown ether moieties formed a bridge (or a cap) above the porphyrin plane, attached to the macrocycle through two β-pyrrolic positions [40]. The ditopic receptor **9** was constructed to bind a cationic entity in the crown ether-like cavity and an anion in the region close to the metalloporphyrin core (Figure 7). The studies showed that the cation binding in the hovering crown pockets of **9-Zn** and **9-Cu** included, but was not limited to, alkali metal cations, transition metal cations, and alkylammonium guests. The binding constants *K*_a_ in CHCl_3_/MeOH 9:1 determined for **9-Zn** complexes with [NH_3_(CH_2_)NH_2_]^+^, [NH_3_(CH_2_)_2_NH_3_]^2+^, [NH_3_(CH_2_)_3_NH_3_]^2+^, and [NH_3_CH_2_CH_3_]^+^ equal to 8.3·10^3^, 6.2·10^3^, 8.7·10^3^, and 7.7·10^2^ M^−1^, respectively, were reported. Based on the fluorescence quenching experiments the formation of coordination compounds of copper(II), iron(II/III), manganese(II), nickel(II), and cobalt(II) with **9-Zn** and **9-Cu** was demonstrated. The emission quenching was rationalised considering the binding of the transition metal within the crown ether cavity. No quenching was observed upon the addition of sodium(I), zinc(II), magnesium(II), and barium(II) [106–109]. Association constants of the reported host–guest complexes showed similar values to those of diazacrown ethers [110–111].

**Figure 8:**
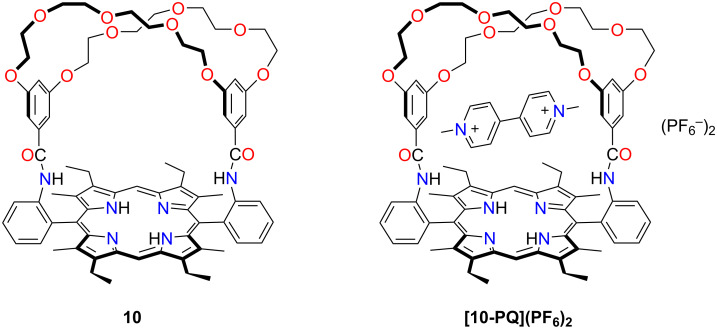
The capped porphyrin **10** and complex **[10-PQ](PF_6_)_2_**.

Johnston and Gunter presented a crown ether-capped porphyrin receptor **10**, which showed unexpected binding affinity towards a dipyridinium cation (Figure 8) [41]. Upon complexation, the guest was sandwiched between the porphyrin and crown ether macrocycles. The work showed a 1:1 complex **[10-PQ^+^](PF_6_)_2_** formation between the electron-poor bipyridinium guest and hybrid macrocycle **10**, indicating a relatively strong attraction between the two parts.

**Figure 9:**
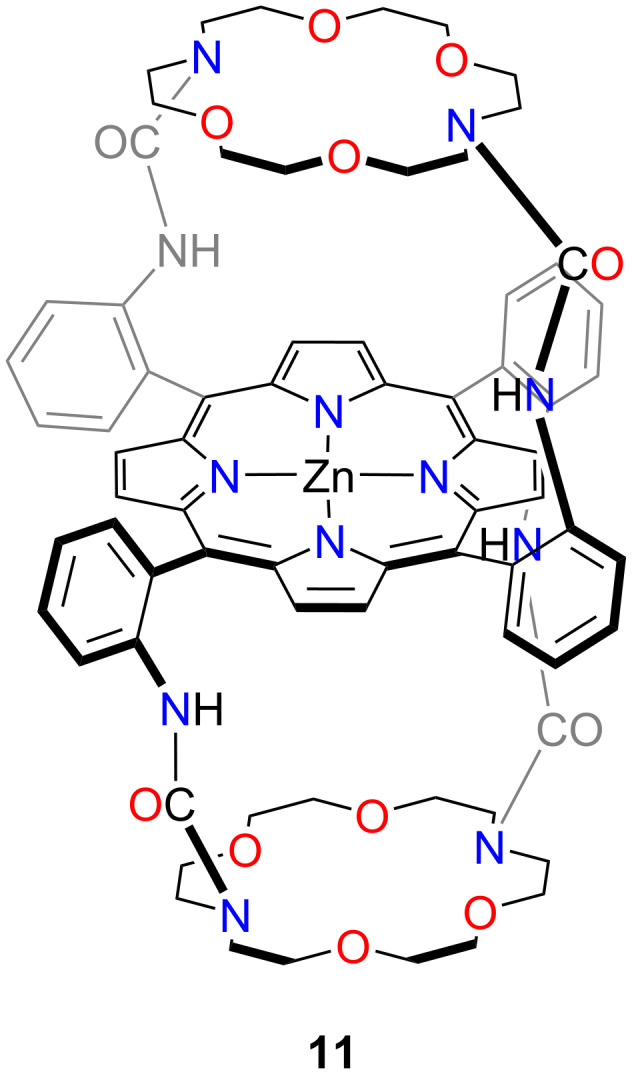
The double-capped porphyrin **11**.

Boitrel developed a crown ether-double-capped zinc(II) porphyrin **11** (Figure 9) [42]. The tritopic receptor incorporated symmetrically positioned diaza-crown-6 units above and below the central porphyrin core. The metalation of the tetrakis(*o*-aminophenyl)porphyrin (TAPP) core with Zn(II) was crucial to achieving the final strapping reaction, affording **11**. The presence of the diaza-crown-6 caps resulted in the *meso*-bridge carbon atoms being slightly pulled out of the porphyrin plane, causing **11** to adopt a ruffled conformation. The penta-coordinated Zn(II) contained an axially bound H_2_O. The water molecule was stabilised by hydrogen bonding to the diaza-crown-6 core.

Over the years, significant advancements have been made in crown ether-capped porphyrins, demonstrating their versatile applications in host–guest chemistry, multitopic receptor design, and cation sensing [23,88,90]. Several studies have focused on developing novel crown ether-appended porphyrins with tailored caps, enabling efficient encapsulation of guest molecules through host–guest interactions [47,81,112]. They have shown promise as heme models and multitopic receptors, exhibiting selective binding towards different cationic species [48,87,113]. Additional efforts have been directed towards designing and synthesising alkali and other cation sensors based on capped crown ether porphyrins, providing enhanced sensitivity and selectivity for specific cations [114–119].

Sessler and co-workers reported on the synthesis of an expanded capped porphyrinoid [120]. The macrocycle incorporated the sapphyrin framework and was demonstrated to act as a ditopic receptor for ammonium fluoride binding cations in the crown ether pocket and fluoride interacting within the expanded porphyrin cavity.

### Crowned Schiff porphyrinoids

**Figure 10:**
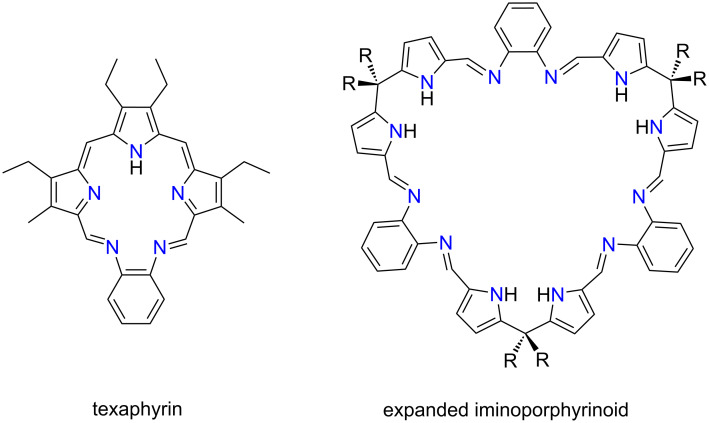
Selected examples of iminoporphyrinoids [58,122].

The distinct group of crown ether–porphyrin hybrids are macrocycles wherein two architectural segments, i.e., an oligo(ethylene glycol) chain and a pyrrole-embedding unit**,** e.g., dipyrrin, tripyrrane, are merged into a single macrocyclic framework. The earliest examples of such systems included Schiff porphyrinoids, a class of macrocyclic compounds incorporating an imine linkage connecting different parts of the macrocyclic skeleton (Figure 10) [30]. This class of compounds has received significant attention due to their intriguing dynamics [33,121] and excellent metal-binding properties [67,122–124].

#### Accordion porphyrins and tripyrrane-crown ether hybrids

**Scheme 4:**
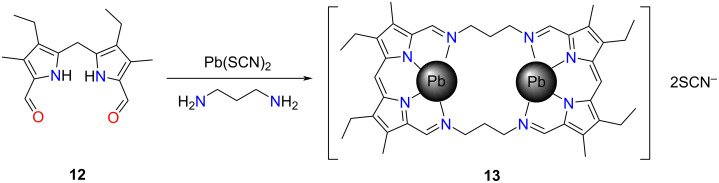
The synthesis of **13**.

The major contribution in the field of iminoporphyrinoids dates back to 1984 when Bowman-James and co-workers demonstrated a facile synthesis of the so-called accordion porphyrins (Scheme 4) [52]. The latter incorporated two dipyrromethane/dipyrromethene units connected through iminoalkyl bridges. The architecture of the macrocycles, and their anticipated dynamic behaviour, wherein two dipyrromethene parts can come closer or further due to the flexibility of the alkyl linker, is reminiscent to that of the action of an accordion. The compounds possessed a large and flexible cavity that could accommodate various guest molecules and metal ions, making them intriguing hosts as well as ligands for transition metals [54]. Several studies have reported on the synthesis and characterisation of accordion porphyrins with various linkers [54,125]. Their formation typically relied on a template synthesis approach [126–127]. The first reported accordion porphyrin was synthesised as a binucleated lead(II) complex **13** (Scheme 4) [52]. The reaction of diformyldipyrromethane **12**, lead(II) thiocyanate, and 1,3-diaminopropane yielded **13** selectively. Later, Bowman-James and co-workers introduced a series of binucleated accordion porphyrins differing in the linkers connecting two parts of the dimeric macrocycle exploiting the barium(II)-templated reactions [53]. The coordination chemistry of accordion porphyrins was investigated, resulting in the formation of lead(II), zinc(II), and copper(II) binuclear coordination compounds [54].

**Scheme 5:**
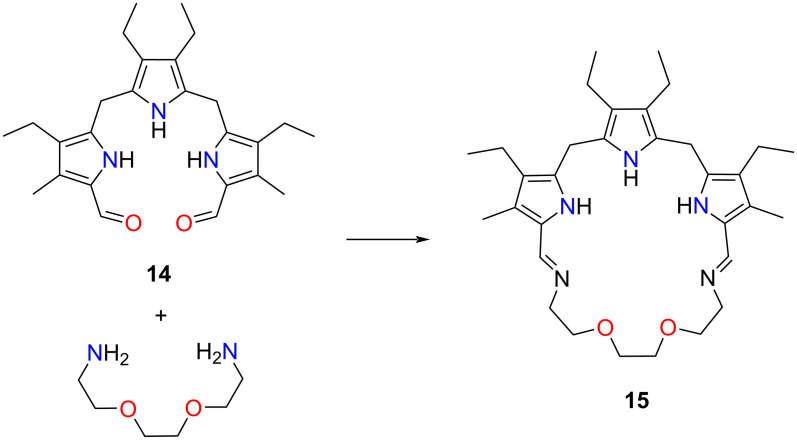
Tripyrrane-based crown ether-embedding porphyrinoid **15**.

The synthetic methodology developed by Sessler allowed to generate a variety of expanded Schiff porphyrinoids [30,128] and texaphyrins [57,60,129]. The group has also presented a crown ether-expanded Schiff porphyrinoid synthesis [56]. Compound **15** incorporated a tripyrrane unit merged with an oligo(ethylene glycol) chain through imine linkages (Scheme 5). The hybrid comprised a core shared amongst a tripyrrane building block and a segment of crown ether linked through imine bonds. Since compound **15** contained an unoxidised tripyrrole subunit, the character of the molecule can be compared to that of porphyrinogens. During the synthesis, a free base or a protonated form of the macrocycle **15** could be obtained.

#### Pacman calixpyrrole-crown ether hybrids

**Figure 11:**
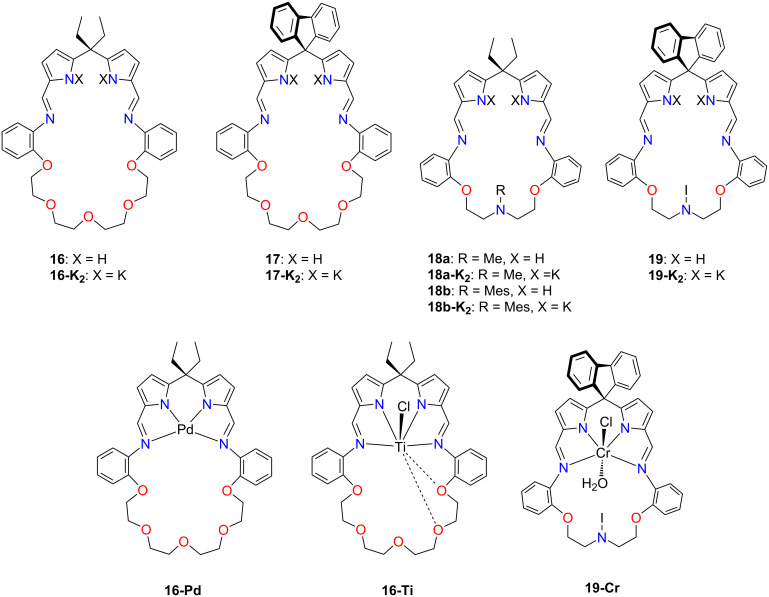
Macrocycles **16–19** and their coordination compounds.

Love and co-workers have developed an alternative approach toward porphyrinoid-crown ether hybrids [66–67]. Replacing the tripyrrane moiety with a *meso*-disubstituted dipyrromethane allowed the creation of a series of macrocycles **16**–**19** differing in the dimensions and heteroatoms within the cavity (Figure 11). The synthesis of these compounds involved the condensation of a *meso*-disubstituted dipyrromethane with diamines incorporating the crown ether/azacrown segment in the presence of boron trifluoride diethyl etherate as a catalyst [66].

**Scheme 6:**
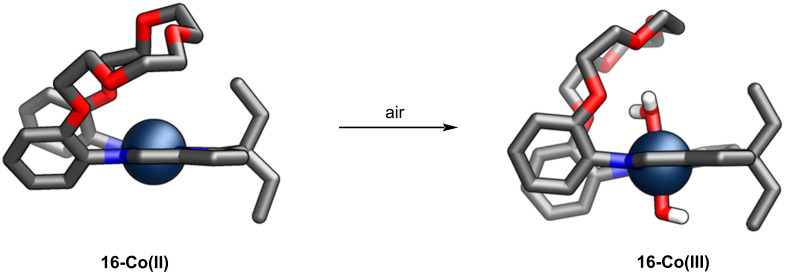
The flexibility of **16-Co** [66].

The treatment of compound **16** with potassium hydride yielded **16-K_2_**, a suitable precursor for coordination compounds. The following transmetallation with cobalt(II) produced an intriguing Pacman-like coordination compound **16-Co(II)** (Scheme 6) [66].

**Figure 12:**
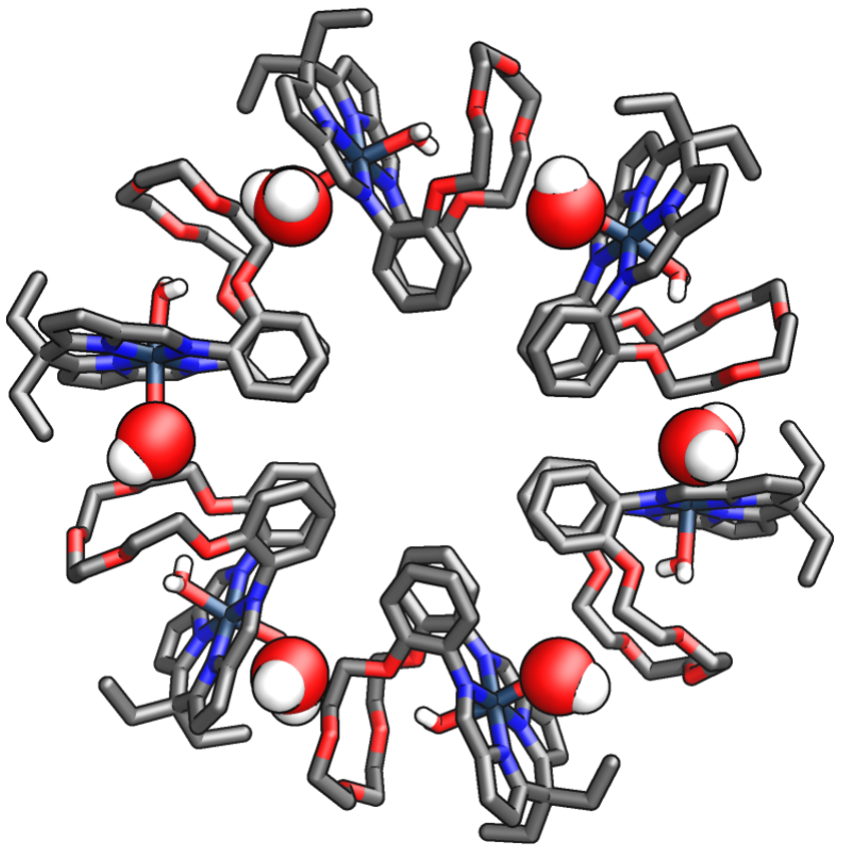
Hexagonal wheel composed of six **16-Co(III)** monomers [66].

The spontaneous oxidation of cobalt(II) to cobalt(III) resulted in modifying the cobalt cation coordination sphere from a distorted-square planar to an octahedral. The water molecule and the hydroxide anion occupied ligand positions on both sides of the median central-core plane, hydrogen bonding to the flexible crown ether part of **16** (Scheme 6). The intramolecular flexibility of compound **16** allowed for tightening/loosening of the cleft, accommodating the axially-positioned water molecule on the cobalt(III) centre. The latter assembled into a remarkable hexagonal wheel architecture when exposed to air in THF, as evidenced by XRD (Figure 12).

The hexagonal wheel **[16-Co(III)]_6_** was stabilised by intramolecular hydrogen bonding between the water molecule bound to the cobalt(III) outside the Pacman cleft and the crown ether part of the adjacent molecule.

**Scheme 7:**
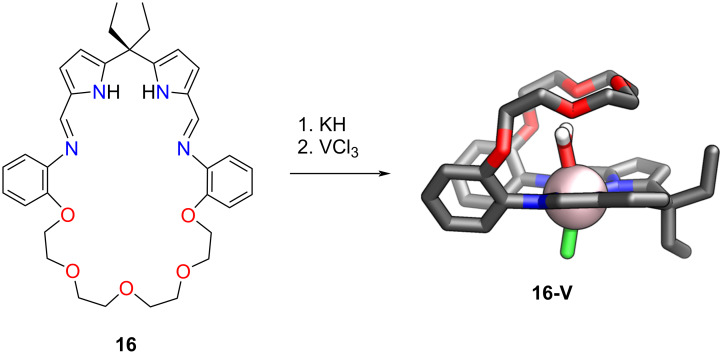
The synthesis of **16-V** [67].

The type and properties of the coordination compound formed from **16–19** depended strongly on the size and structure of the crown ether part of the molecule and the transition metal [67]. Palladium(II) compounds **16-Pd–19-Pd** exhibited conformations with the characteristic Pacman clefts, resembling their postulated solution structures and typical square-planar geometry around the palladium(II) centres. Titanium(III), vanadium(III), and chromium(III) complexes were synthesised through salt elimination reactions between in situ generated **16-K_2_–19-K_2_** and MCl_3_(THF)_3_ yielding **16-Ti**, **18a-Ti** (structure not shown), **16-V** (Scheme 7), **18a-V** (structure not shown), and **19-Cr** [67].

The Ti(III)-incorporated **16-Ti** and **18a-Ti** exhibited paramagnetic properties; their identity was confirmed through elemental analysis and mass spectrometry. Similar to the Ti(III) complexes, the V(III) compounds **16-V** and **18a-V** demonstrated paramagnetic features. The V(III) cation resided in a distorted octahedral environment, slightly outside the cleft, and was bound by two imine and two pyrrolic nitrogens (Scheme 7) [130]. The water molecule was accommodated within the cleft of the Pacman-shaped macrocycle, occupying the axial position. The V–O bond length of 2.111(3) Å indicated the coordinated entity's V(III)–OH_2_ character. The water molecule in the cleft was further stabilised by hydrogen bonding to the ether oxygens, emphasising the structural motif's ability to stabilise guest molecules through primary and secondary sphere bonding interactions. Compound **19-Cr**, similarly to **18a-Ti** and **18a-V**, also exhibited paramagnetic features. The solid state structure demonstrated the complex featuring a Cr^3+^ cation bound in a distorted octahedral fashion, with a water molecule accommodated within the molecular cleft. The Cr(III) resided in the N_4_-plane and was coordinated by two imine and two pyrrolic nitrogens. The water molecule was stabilised by hydrogen bonding to nearby oxygens from the crown ether part and to the pendant nitrogen in the middle of the ether chain of **19-Cr**.

The reaction between in situ generated **17-K_2_** or **19-K_2_** and CoCl_2_ produced paramagnetic cobalt(II) complexes **17-Co** or **19-Co** [67]. The XRD analysis of **17-Co** revealed a molecular structure with distorted octahedral Co(II) coordinating water and hydroxide ligands. Compound **19-Co** (structure not shown) retained the cobalt(II) oxidation state with a water molecule within the cleft. The XRD analysis of the structures of **19-Co** and **18b-Co** exhibited Pacman conformation. The X-ray molecular structure of **19-Co** provided further insights, showing a square-planar geometry with the cobalt(II) positioned slightly above the N_4_ donor plane. The X-ray structure of **18b-Co** exhibited a similar Pacman motif as its palladium analogue, with the cobalt(II) cation residing in a square-planar environment.

**Figure 13:**
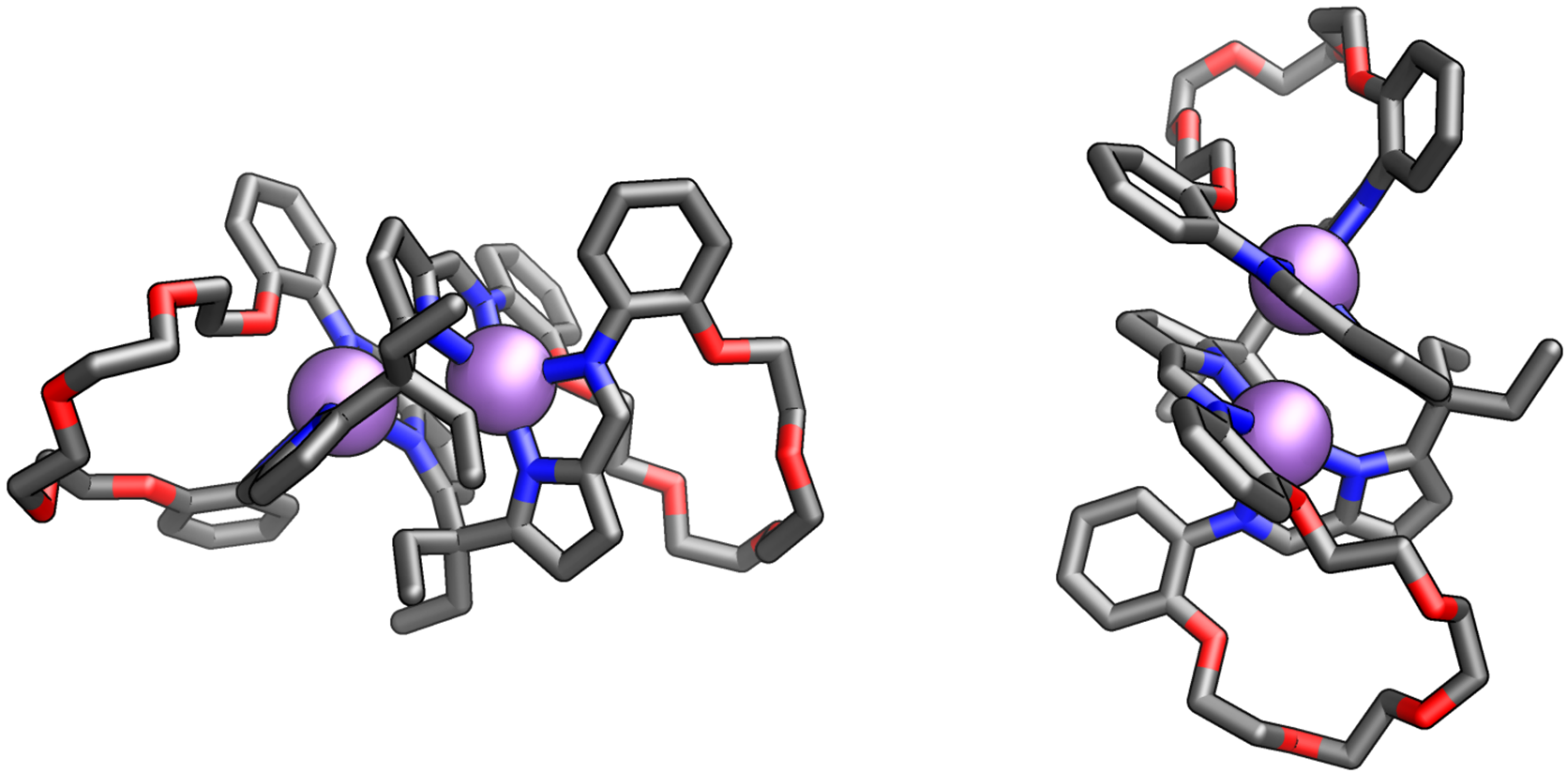
The molecular structure of dimers **[16-Mn]_2_** [67].

The exploitation of a similar synthetic methodology allowed for preparing iron(II) and manganese(II) complexes with similar compositions. Interestingly, by-products incorporating the [2 + 2] macrocycles were isolated from the reaction mixtures targeting **16-Fe, 18a-Fe** and **16-Mn**, **18a-Mn** (Figure 13) [67].

The helical geometry of **[16-M]_2_** was attributed to the inherent flexibility of macrocyclic ligands, as demonstrated by X-ray molecular structures. The helicates consisted of two metal centres, namely cobalt, manganese, or iron, each coordinated by two nitrogens and oxygen donors of the ligand. The metal centres adopted distorted octahedral geometries with slight deviations from the N_3_O plane. An intriguing feature of the helicates was their short metal–metal separation (3.151 Å [Fe], 3.521 Å [Mn], and 3.104 Å [Co]) enabled by the flexibility of the ligand incorporating the sp^3^-hybridised *meso*-carbons.

#### Crownphyrins and similar systems

**Scheme 8:**
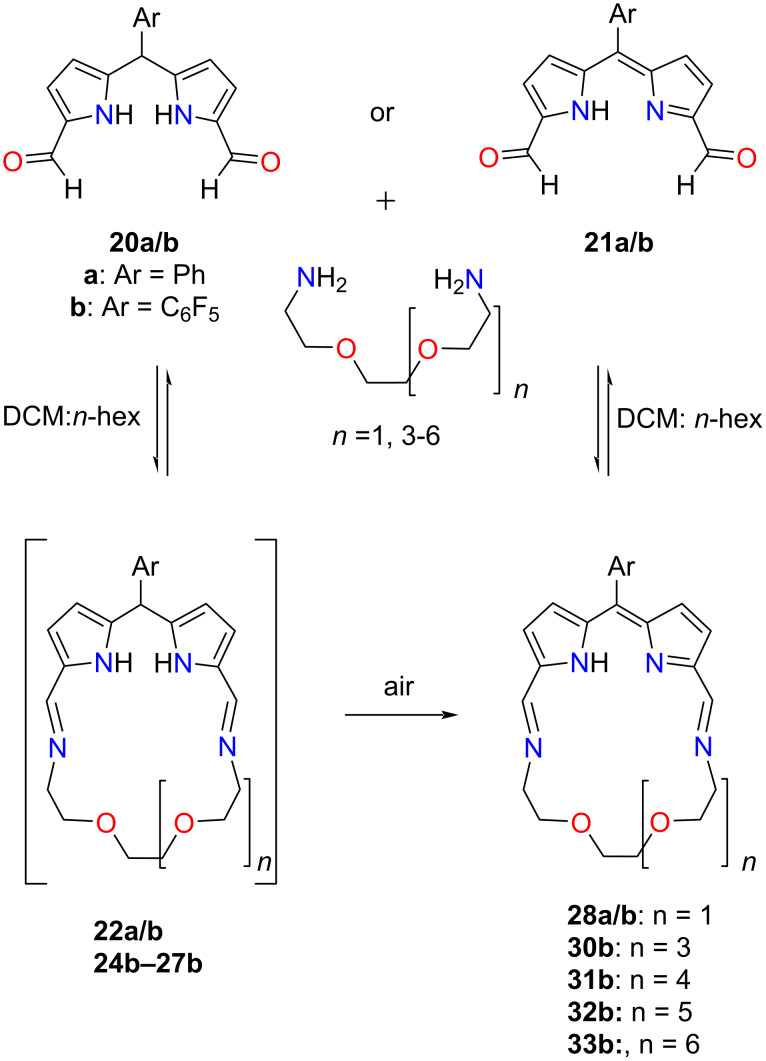
Synthesis of crownphyrins **28–33**. Compounds **23a/b** and **29a/b** were obtained from 4,7,10-trioxa-1,13-tridecanediamine.

**Figure 14:**
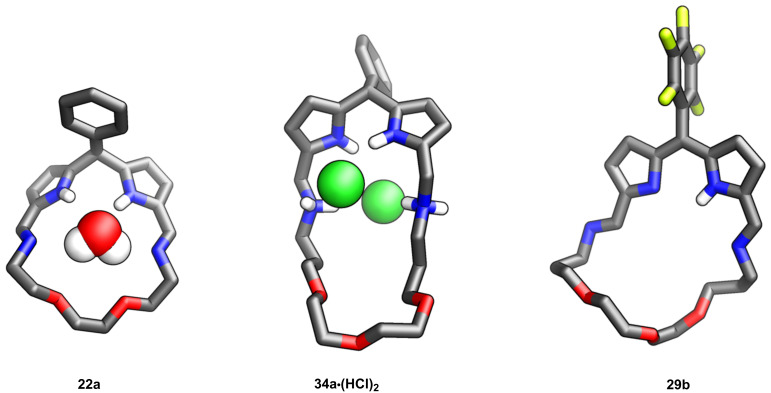
The molecular structures of **22a**, **34a·(HCl)_2_**, and **29b** [69].

In 2022 our group reported on the synthesis of crownphyrins **28–33**, namely macrocycles that combine the structural facets of crown ethers and porphyrins (Scheme 8) [69]. In contrast to the tripyrrane **15** and *meso*-dialkyldipyrromethane-based macrocycles **16–19** reported by Sessler and Love, the crownphyrinogens **22–27** exhibited a notable distinction as they could readily undergo oxidation to yield the corresponding crownphyrins **28–33**, incorporating a dipyrrin unit. The synthetic pathway towards crownphyrins is straightforward and relies on a one-pot reaction between diformyldipyrromethane **20a/b** and a diamine which introduces the ether segment of desired length (Scheme 8). The procedure is relatively facile and can provide high yields of the target macrocycles, requiring minimal work-up. Depending on the starting material and the aryl group in the *meso*-position of **20**, the dipyrromethane-incorporating crownphyrinogens **22a** or crownphyrins **28–33** could be isolated. Upon reduction, the macrocycles demonstrated chloride binding (**34a·(HCl)_2_**; Figure 14).

**Figure 15:**
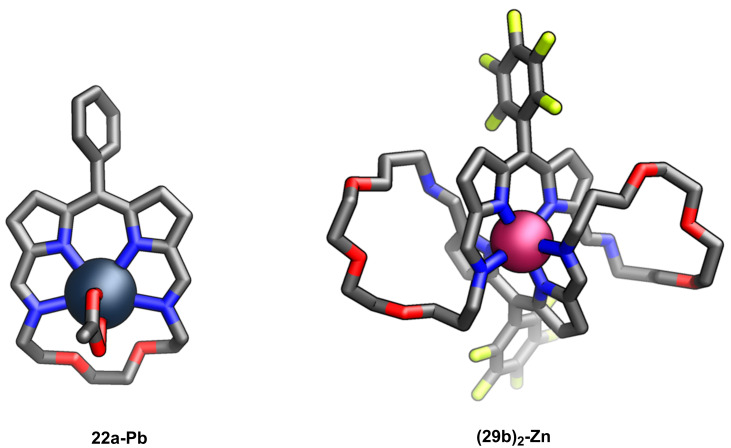
Molecular structures of **22a-Pb** and **(29b)_2_-Zn** [69].

The presence of a dipyrrin unit within the crownphyrin molecules rendered them intriguing macrocyclic ligands. The reaction of **22a** with lead(II) acetate provided a monomeric complex **22a-Pb**, wherein the metal centre is coordinated through four nitrogen donors and only weakly interacts with two oxygen atoms of the ether segments (Figure 15).

**Scheme 9:**
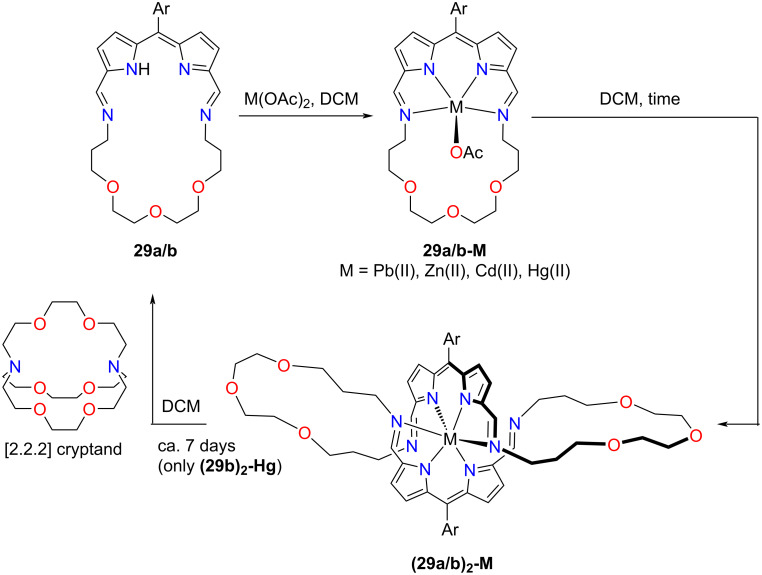
Reactivity of **29a/b**.

Once crownphyrins reacted with geometrically more demanding than Pb(II) metals, i.e., zinc(II), cadmium(II), and mercury(II), they initially formed analogous, monomeric complexes **29a/b-M** (Scheme 9). However, when left in the solution, **29a/b-M** spontaneously transformed into **(29a/b)_2_-M**, incorporating a dimeric macrocycle.

Notably, the transformation of the macrocycle represented an unprecedented [1 + 1] to [2 + 2] expansion of iminopyrrole macrocycles. The formation of **(29b)_2_-Hg** was reversible – its reaction with [2.2.2]cryptand resulted in the removal of mercury(II) and the contraction to **29b**.

**Scheme 10:**
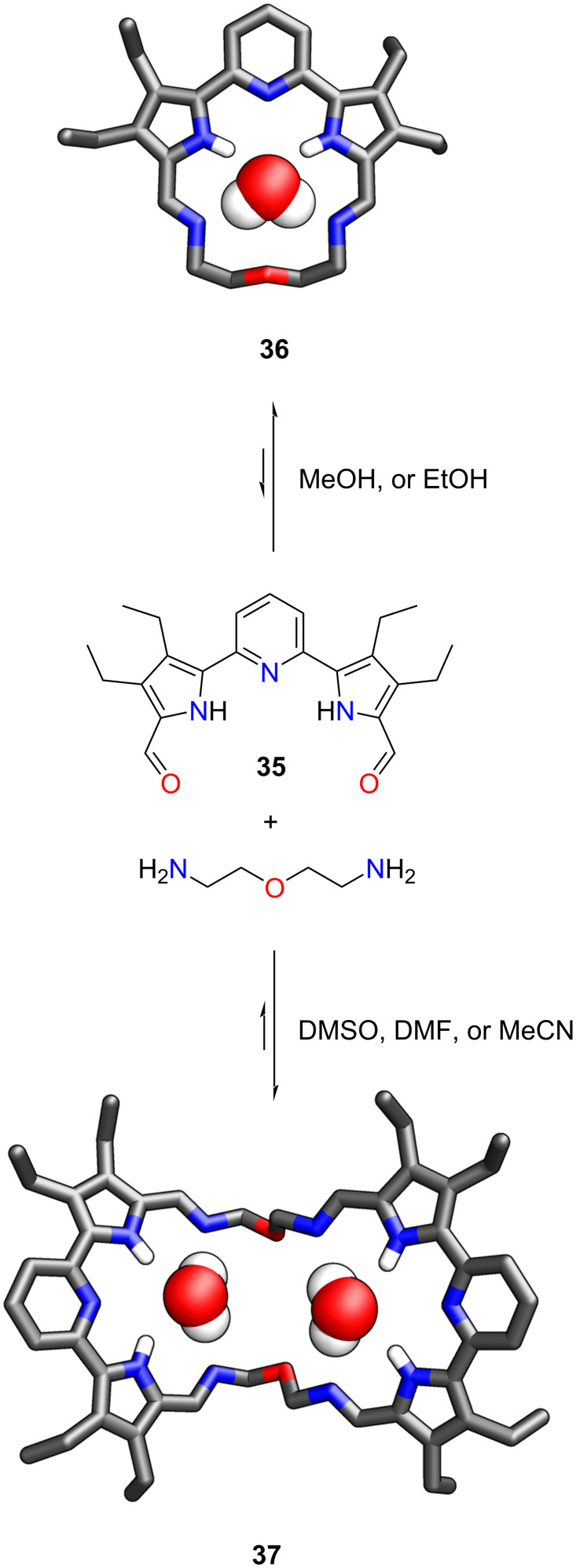
Synthesis of **36** and **37** [131].

Recently, Sessler and co-workers synthesised a new macrocycle **36** exploiting a pyridine-bridged dipyrroledialdehyde **35** (Scheme 10) [131]. The compound demonstrated interesting, reversible, solvent-directed macrocycle-to-macrocycle interconversions. The transformations between the [1 + 1] **36** and [2 + 2] **37** macrocycles were governed by the solvent. The smaller monomeric **36** was obtained as the major product in chloroform, methanol, and ethanol. The conversion to a dimer **37** was achieved using *N*,*N*-dimethylformamide, dimethyl sulfoxide, or acetonitrile. Interestingly, the dissolution of compound **37** in CHCl_3_, MeOH, or EtOH resulted in the interconversion to **36** within 1–2 days, as evidenced by ^1^H NMR spectroscopy.

**Scheme 11:**
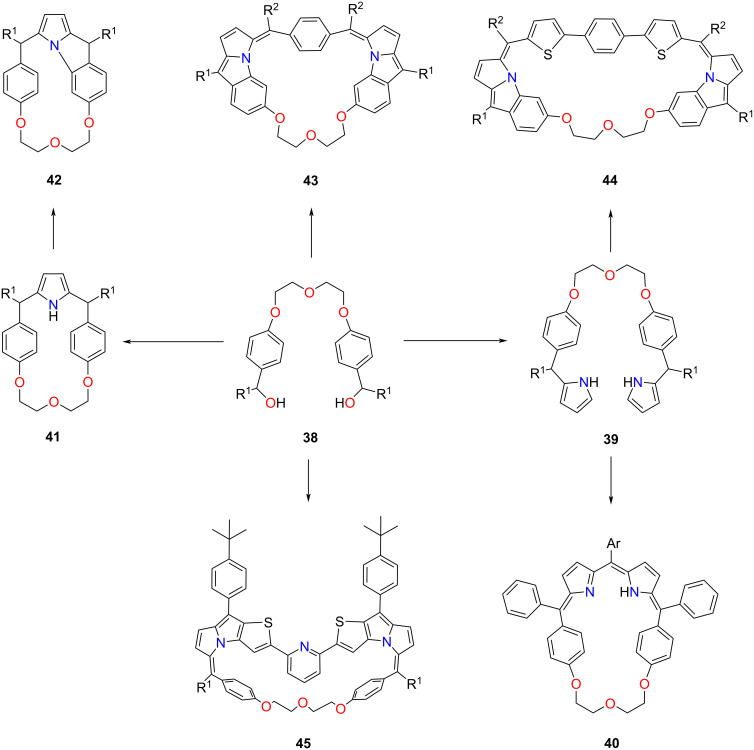
Synthesis of **40–45**.

The Ravikanth group has developed an alternative approach towards crown ether–porphyrin hybrids. They have introduced the crown ether segment by exploiting a dicarbinol **38**, which, once subjected to the reaction with pyrrole, formed **39** [68]. The latter reacted with arylaldehydes under acidic conditions yielding **40** (Scheme 11). Protonation of **40** with trifluoroacetic acid (TFA) resulted in a cationic **40-H^+^**. Cyclic voltammetry and differential pulse voltammetry were performed to investigate the electrochemical properties of **40-H^+^**. The cation exhibited two reversible oxidations and two to three reductions. The redox potentials were influenced by the aryl group at the *meso*-position of the dipyrrin moiety. Compound **40** was tested for sensing metal ions, and while no significant changes were observed with most cations, the addition of Cu(II) resulted in a colour change. UV–vis spectroscopy and mass spectrometry confirmed the 1:1 copper(II) complex **40-Cu** formation proving that **40** acts as a colourimetric sensor.

The reaction of **38** with pyrrole in the presence of BF_3_:Et_2_O resulted in **41** incorporating a single pyrrole ring [132]. The attempted oxidation with DDQ afforded fused macrocycle **42** (Scheme 11). The X-ray molecular structure of **42** revealed a distorted ruffled molecule with a macrocycle incorporating a pyrroloindole subunit formed through the fusion between the *para*-phenylene ring and the pyrrolic nitrogen. **42** demonstrated fluorophore behaviour with relatively large fluorescence quantum yields of 10–20% and singlet state lifetimes of 1.70–2.50 ns. An apparent colour change was observed upon treatment of **42** with AgSbF_6_ and CuCl_2_, indicating radical cation formation **42**^•+^. ESR spectra and coulometric oxidation experiments further supported the presence and stability of the radical species.

The reactions of **38** with a pre-functionalized dipyrromethane moiety provided expanded carbaporphyrinoids incorporating flexible oligoethylene glycol segments **43** (Scheme 11) [133]. The series of fused macrocycles **43** were obtained in 10–15% yield. The formation of **43** was additionally evidenced by ^1^H NMR spectroscopy, and the macrocycle **43** was analysed by single crystal XRD. Compound **43** formed stable cation radicals upon adding different oxidising agents, such as AgSbF_6_, TFA, and CuCl_2_. The cation radicals showed relative stability and remained undeteriorated on air for over a week.

The crowned porphyrinoids incorporating two pyrroloindole units **44** were also synthesised (Scheme 11) [134]. The electrochemical studies demonstrated low oxidation potentials, and similarly to previously described systems incorporating a single pyrroloindole unit, compound **44** underwent single-electron oxidation forming stable cation radicals. Ravikanth and co-workers have also demonstrated the crowned fused expanded porphyrinoids incorporating a pyridine moiety [135]. Macrocycles **45** were obtained in 5–10% yield from the condensation of **38** with the corresponding pyridine-based dipyrromethane analogue. Compound **45** exhibited a unique structural arrangement, with the pyridine ring and two thiophenes inverted and fused with two pyrrole nitrogen atoms. The macrocycles exhibited facile oxidations, indicating their electron-rich nature, and demonstrated selective sensing of Cu^2+^ ions.

## Conclusion and Outlook

The construction of new macrocycles has been a driving force for the development of various areas of supramolecular chemistry. Porphyrins and crown ethers continue to play a significant role in these studies. As a result of combining structural motifs from both classes of archetypal macrocyclic compounds, a series of fascinating molecular objects representing hybrid connections have been obtained. Since the 1970s, these compounds have been actively investigated for their use as unconventional ligands in coordination chemistry, multitopic receptors capable of binding guests in cavities of different chemical natures, and chemosensors enabling the selective detection of various analytes.

**Figure 16:**
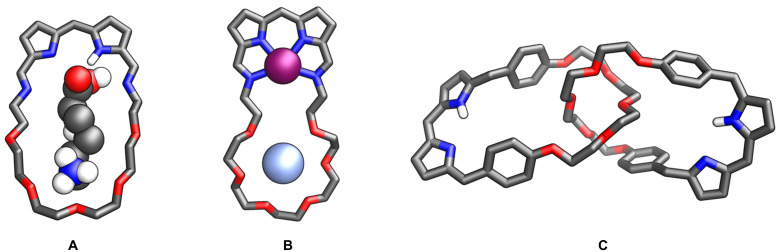
Potential applications of porphyrin-crown ether hybrids.

Although several intriguing applications of crowned porphyrins have been elaborated, the potential of macrocycles encompassing pyrrole and crown ether motifs in a single macrocyclic framework is yet to be revealed. One can anticipate that an interesting system will be created by exploiting them for molecular recognition (Figure 16A). The dual nature of hybrids offers promising prospects, with a coordination pocket enabling selective binding of organic molecules such as natural and non-natural amino acids, hormones, neurotransmitters and other biomolecules. In such complexes, the fragment originating from the porphyrinoid could form hydrogen bonds with a carboxyl group, while the crown ether cavity would allow interaction with the protonated amine group of an amino acid molecule. The choice of macrocycle size could enable the recognition of different biomolecules. Another attractive research area is the synthesis of dinuclear coordination compounds, allowing for the stabilisation of two metal centres in close proximity (Figure 16B). Complexes of this kind will exhibit unusual magnetic and spectroscopic properties resulting from the short distance between the metal cations. These molecules could serve as models for enzyme active centres and present intriguing catalytic features. Additionally, a separate and equally intriguing group of molecules that can be achieved by taking advantage of the hybrid porphyrin–crown ether compounds includes mechanically interlocked molecules, e.g., catenanes and rotaxanes (Figure 16C). The formation of such compounds would eventually result in the formation of a unique group of three-dimensional ligands with co-existing porphyrin-like and crown ether cavities.

The perspective article has highlighted the wide range of crown ether–porphyrin hybrid systems synthesised and studied over the past few decades. The synthesis and characterisation of various types of crowned porphyrins have been discussed, and their potential to act as precursors for more complex architectures has also been showcased. Finally, the emergence of new classes of hybrids, such as crownphyrins and Schiff-base calixpyrroles, has been discussed, providing some new directions in the field. The continued development of hybrid systems is anticipated to provide exciting opportunities for further explorations and bring many intriguing molecular systems with fascinating applications.
